# ERAS in Cardiac Surgery: Wishful Thinking or Reality

**DOI:** 10.4274/TJAR.2023.231238

**Published:** 2023-10-24

**Authors:** Z. Aslı Demir, Nandor Marczin

**Affiliations:** 1 University of Health Sciences Turkey, Ankara Bilkent City Hospital, Clinic of Anaesthesiology, Ankara, Turkey; 2 Imperial College London, Department of Cardiac Anaesthesiology, London, United Kingdom

**Keywords:** Cardiac patient, cardiac surgery, cardiovascular and thoracic anaesthesia, enhanced recovery after surgery, ERAS

## Abstract

Enhanced recovery after cardiac surgery (ERACS) is a multi-disciplinary approach to improve patient outcomes and reduce complications following cardiac surgery. The aim of ERACS protocol is to optimize pre-operative preparation, reduce surgical trauma, and minimize post-operative stress.The protocol has been shown to improve patient outcomes, including shorter hospital stays, lower rates of complications, and faster return to normal activities. It is important to note that ERACS is a multi-disciplinary approach, and requires close collaboration between surgeons, anaesthesiologists, nurses, and other healthcare professionals to ensure successful implementation. Anaesthesiologists play a crucial role in the ERACS protocol, as they are responsible for the management of the patient’s anaesthesia and pain management during and after surgery. In this paper provide an overview of the ERACS protocol from the perspective of an anaesthesiologist.

**Main Point**

• In this article, the ERAS adventure in cardiac surgery was evaluated from all aspects.

## Introduction

All new initiatives are viewed with skepticism by clinicians. It is always difficult to get out of the ordinary and try to adapt to something new. After all, in addition to ensuring patient satisfaction, we in the medical profession are bound to never compromise on the motto of “premum non nocere”. And it is undoubtedly true that the shortage of trained personnel, equipment, and high costs are barriers to undertaking brave new beginnings in the struggling health system. However, all of these realities are in fact the main reasons for undertaking new ventures. Enhanced recovery after surgery (ERAS) protocols (Table 1), an exemplar of novel initiatives and the focus of this paper, are journeying down the same difficult path. Since the day they were defined, the measures have faced a steady barrage of criticism. Detractors have accused the protocols of a variety of shortcomings, including endangering patient safety because of the high costs involved and not having any effect on patient results in general. They have even claimed that the favorable outcomes are the result of psychological reflection of patients’ inclusion in a special program (Hawthorn effect).^1^ However, studies emphasizing the positive aspects of ERAS applications on patient outcomes along with shortened hospital stays are not to be underestimated.^2,3,4^

The fact that cardiac patients have more comorbidities as well as cardiorespiratory system problems necessitated a cautious approach to the concepts of early recovery, so ERAS, which was first defined in for colon surgery in 1997, could only actively implemented in cardiac surgery practice in 2016.^5^ The implementation of ERAS protocols, which includes many steps starting from the preoperative period and covering the intraoperative and postoperative period, requires the joint and harmonious action of many departments, including the patient, dietitian, physiotherapist, surgeon, anaesthesiologist, intensivist, perfusionist, and nurse. There are many steps in the pre-intra and postoperative periods of the enhanced recovery after cardiac surgery (ERACS) protocols. Which one is the most important step over the entire perioperative process is not clear; carefully preparing the patient for surgery, early extubation, or early removal from the intensive care unit (ICU)? Which is the hardest step to implement? Should ERAS be applied only to patients in good general condition?

With the idea that the answers to many such questions can be determined according to the internal dynamics and resources of each hospital, implementation of institutional ERACS protocols began in Ankara Bilkent City Hospital, Cardiac Surgery and Anaesthesia Clinics in 2020. In the protocol, applied in all surgeries except for emergency surgery, each patient preparing for surgery was informed approximately 1 month before the surgery about the procedures involved, and their smoking/alcohol use, anemia, HbA1c, and their nutritional status were evaluated and comorbidities strictly optimized. However, it is not always possible to examine and evaluate the cardiac surgery patient one month before the surgery, as they may not be able to wait another month. Given that patients with shorter preparation time constitute the majority of all cardiac surgery patients, it is important to note that ERAS protocols are described in the literature as applicable in emergency surgeries and positively impact the results.^6,7^

In emergency surgeries the “preoperative” column of the ERAS steps is bypassed out of necessity. Although this skipped column contains important protocol components, from prehabilitation to smoking cessation, and comorbidity optimization to blood glucose-HbA1c correction, positive surgery outcomes and short hospital stays can still be achieved. The question then comes to mind: is the preoperative ERAS column “expendable”? Would a refined “shorter list”, not including all ERAS components, suffice? Conversely, there are publications describing the importance of prehabilitation and protesting that only 5 out of 13 ERAS guidelines mention prehabilitation. Robust randomized controlled trials are required to clarify this issue.

It is one of our surprising findings that 37% of the patients who underwent detailed nutritional examinations in the preoperative period within the scope of our protocol were found to have malnutrition. The chronic inflammatory state, which is the likely etiology of comorbidities such as hypertension, chronic obstructive pulmonary disease, and diabetes mellitus accompanying cardiac disease, also negatively affected nutrition. Following this determination, the nutrition team ensured that these patients were included in a special nutrition program.

The relationship between ERAS and diabetic patients is an ongoing focus of research. Although the relationship between gastroparesis and diabetes in terms of passive regurgitation and pulmonary aspiration was investigated and was not found to be risky,^8,9,10^ it has been observed that different management is required in terms of hyperglycemia. In diabetic (type 1 or type 2) patients, in whom we applied 50 g (12.5%) oral maltodextrin carbohydrate loading in 400 mL liquid in the preoperative 2^nd^ hour, which is included as a weak recommendation in the ERACS protocols,^11^ excessive blood sugar elevations were encountered with the start of surgery. Many ERACS protocols do not include diabetic patients in their programs, but we think that a careful protocol can be applied to risky patients as well. As current ERACS protocols do not offer specific recommendations regarding this subgroup of patients, the need for close monitoring of blood glucose levels and management of anti-diabetic medication and case based clinical judgment is evident until more evidence has been available.^12^ As such, in our practice, diabetic patients were given carbohydrate rich clear fluids 3 hours instead of 2 hours, preoperatively. By applying a different insulin regimen in the perioperative period, the problem of hyperglycemia was largely resolved. No pulmonary aspiration complication was encountered in patients taking carbohydrate loading, including patients undergoing transesophageal echocardiography.

One of the drugs recommended for premedication in ERACS protocols is 150-300 mg oral pregabalin.^13^ Since severe disorientation was found in patients who were given 300 mg of pregabalin in our protocol, this dose was changed to 150 mg or less. One of bilateral erector spina plan block, serratus anterior plane block, or parasternal block was applied to the patient before anaesthesia, for intraoperative and postoperative analgesia. Anaesthesia is provided by an inhalation agent or propofol infusion, to which short-acting opioid (remifentanil) analgesia is added. The bispectral index (BIS) values are kept between 40-45 throughout the operation in order to prevent anaesthetic-related postoperative delirium and cognitive impairment. Further decrease in BIS is not allowed and midazolam was avoided. At the same time, by monitoring bilateral cerebral oxygenation with near infrared spectroscopy, many vital situations such as prevention of cannula malposition, optimization of pump flow, and detection of critical blood pressure values in severely hypertensive patients are directed. Meticulous glucose monitoring to keep blood glucose <180 gr dL^-1^, patient blood management and cardiopulmonary bypass (CPB) management with retrograde autologous prime method are routinely performed. Goal directed fluid therapy was managed with hemodynamic monitors, especially from the end of the surgical procedure and the weaning from CPB. In the postoperative period, patients were extubated within 6-8 hours, early mobilization and feeding were started, and strict nausea-vomiting prophylaxis was performed. Within the scope of fast-track and ERACS protocols, extubation is recommended within 6 hours postoperatively.^13,14,15^ On the other hand, it is claimed that there is no significant difference in the duration of ICU and hospital stay between the patients extubated in the first 6 hours and those extubated between 6-12 hours.^15^ Although our patients actually met the extubation criteria comfortably from the 3^rd^-4^th^ hour, unfortunately, conservative teams in the ICU became a barrier to earlier extubation. However, for a relatively newcomer implementation, these are situations where progress can be made over time. In the postoperative period, in addition to regional methods, analgesia management is provided with tramadol and paracetamol. In our clinic, non-steroidal anti-inflammatory drugs are not administered to cardiac patients due to the possibility of prothrombosis, bleeding, and renal damage. In addition, there is no routine steroid administration for anti-inflammation due to insufficient evidence about its effects. In the postoperative period, regular delirium, cognitive function, and Acute Kidney Injury screening are performed (additional data: Ankara City Hospital, ERACS Protocol).

Potential barriers to the implementation of a cardiac surgery ERAS Program are threefold: patient-related factors such as unrealistic expectations, poor health literacy, poor patient engagement, and comorbidities; staff-related factors such as doctor and staff preferences, lack of information, and poor communication and cooperation; and finally system related factors such as lack of medicine, equipment and personnel, lack of leadership, and financial problems.^13^

Our initiative, which is Turkey’s first ERACS implementation included 400 patients. It is difficult, however, to say exactly which is the most important step of the ERACS protocol, which steps can be sacrificed, and which are indispensable. In the intraoperative period, in which we, as anaesthesiologists, are most involved, the improvement in patient outcomes - with the use of opioid-reduced methods and regional techniques, the realization of blood management strategies in full harmony, and the intervention of the patient with more monitors - are quite satisfactory. In addition, early extubation may be the intervention which shows that everything is going well with the patient in the early postoperative period. This stage is a critical step that indicates the adequacy of hemodynamic stabilization, cardiorespiratory status and major cerebral functions, and is a sign of early ICU exit. ICU stay or length of hospital stay is often used as a measure of success for ERAS and fast-track protocols, however, length of stay in the healthcare system is more directly related to managerial and organizational issues than to patient recovery. When the ERAS guidelines are examined, the level of evidence and power of anaesthesia management practices are higher.^13,16^ The anaesthesiologists plays the most critical role in the management of ERAS pathways by acting as a bridge between the perioperative periods.

Before undertaking the implementation, we considered that the patients would have difficulty following the rules and that the responsible personnel (dietician, physiotherapist, nurses..) would perceive this as an extra routine workload and disrupt the flow (because no one receives payment for implementation applications). However, both the patients and the responsible staff contributed very enthusiastically to the protocol and this prediction turned out to be incorrect. The most difficult obstacle in the initiative was the persuasion of the intensive care team (doctor and nurse) to the idea of early extubation. Although the concept of fast-track extubation has a long history, and although patients already met all the criteria for extubation, staying intubated somehow made the ICU team feel more comfortable and safe. However, over time, the intensive care team realized that many patients were intubated unnecessarily.

Another point of view regarding ERACS was that it would be more appropriate to apply the protocol only to well-being, low-risk, selected patients. However, considering that good results can be obtained without applying a special protocol to patients in good condition, ERACS might be perceived as an unnecessary effort. However, with the widespread effect of an application that has been found to improve patient outcomes and its application to risky patients, it actually may cause more attention to all patients who require care. Similarly, we observed that the application of ERACS in high-risk patients in critical condition had a positive effect on the outcome.^17,18,19^

ERACS is a procedure that requires multidisciplinary participation. This paper’s aim was to discuss the ERACS protocols from the perspective of anaesthesiologist’s instead of the whole protocol. One of the facts that emerge in this context is that the anaesthetist is a crucial practitioner who not only provides the ideal anaesthetic approach throughout the procedure, but also helps to improve overall perioperative care. When we summarize the initial unpublished design and implementation of an institutional ERACS protocol for cardiac surgery, the results suggest that compliance with a dynamic preanaesthetic preparation and a range of intraoperative evidence-based practices can translate into more comfortable and shorter hospital stays after cardiac surgery. While no difference was found in terms of complications, a cost reduction of up to 24% stands out as an additional result.^20^

## Figures and Tables

**Table 1:**
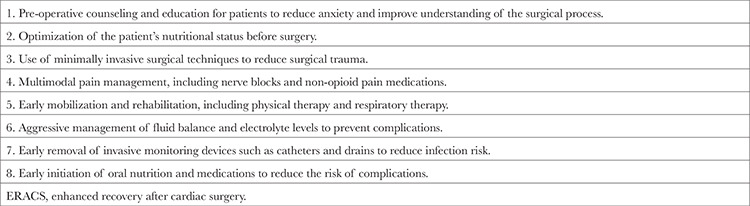
The ERACS Protocol Involves a Number of Interventions, Including
